# How Peer Pressure Shapes Consensus, Leadership, and Innovations in Social Groups

**DOI:** 10.1038/srep02905

**Published:** 2013-10-09

**Authors:** Ernesto Estrada, Eusebio Vargas-Estrada

**Affiliations:** 1 Department of Mathematics & Statistics, University of Strathclyde, Glasgow G1 1XH, UK; 2 Institute for Quantitative Theory and Methods (QuanTM), Emory University, Atlanta, GA 30322, USA

## Abstract

What is the effect of the combined direct and indirect social influences—peer pressure (PP)—on a social group's collective decisions? We present a model that captures PP as a function of the socio-cultural distance between individuals in a social group. Using this model and empirical data from 15 real-world social networks we found that the PP level determines how fast a social group reaches consensus. More importantly, the levels of PP determine the leaders who can achieve full control of their social groups. PP can overcome barriers imposed upon a consensus by the existence of tightly connected communities with local leaders or the existence of leaders with poor cohesiveness of opinions. A moderate level of PP is also necessary to explain the rate at which innovations diffuse through a variety of social groups.

The social group's pressure on an individual—peer pressure (PP)—has attracted the attention of scholars in a variety of disciplines, spanning sociology, economics, finance, psychology, and management sciences^1^,^2^,^3^,^4^. In analyzing PP we should consider not only those individuals directly linked to a particular person, but also those who exert indirect social influence over other persons as well^5^,^6^,^7^,^8^. Although PP is an elusive concept, it can be considered a decreasing function of a given individual's socio-cultural distance from the group. Thus, an individual's opinion may be influenced more strongly by the pressure exerted by those socio-culturally closer to her. Consensus is well documented across the social sciences, with examples ranging from behavioral flocking in popular cultural styles, emotional contagion, collective decision making, pedestrians' walking behavior, and others^9^,^10^,^11^,^12^.

We can model consensus in a social group by encoding the state of each individual at a given time *t* in a vector **u**(*t*). The group reaches consensus at *t* → ∞ when 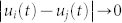 for every pair of individuals, and the collective dynamics of the system is modeled by 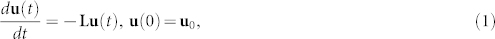
where **L** is a linear operator (Laplacian matrix) capturing the topology of the social network^9^.

Decisions in groups trying to reach consensus are frequently influenced by a small proportion of the group who guides or dictates the behavior of the entire network. In this situation a group of leaders indicates and/or initiates the route to the consensus, and the rest of the group readily follows their attitudes. The study of leadership in social groups has always intrigued researchers in the social and behavioral sciences^13^,^14^,^15^,^16^,^17^. Specifically, the way in which leaders emerge in social groups is not well understood^18^. Leaders may emerge either randomly in response to particular historical circumstances or from the individual having the most prominent position (centrality) in the social network at any time.

## Results

### Emergence of leaders and PP

To capture the influence of PP over the emergence of leaders in social groups, we consider that the pressure that an individual *p* receives from *q* deteriorates proportionally with the social distance between *p* and *q*. The social distance is captured by the number of links in the shortest path connecting *p* and *q*. Mathematically, we model the mobilizing power between two individuals at distance *d* as Δ*_d_* ~ *f*(*d*)^−1^, where *f*(*d*) represents a function of the social distance (see Methods equations (11) and (12)). The collective dynamics of the network under peers' mobilizing effects is described by the following generalization of the consensus model 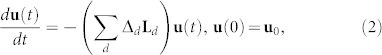
where **L***_d_* captures the interactions between individuals separated by *d* links in their social network, Δ*_d_* ~ 1/*d^α^* where the parameter *α* accounts for the strength of the PP pulling an individual into the consensus.

**Figure 1:**
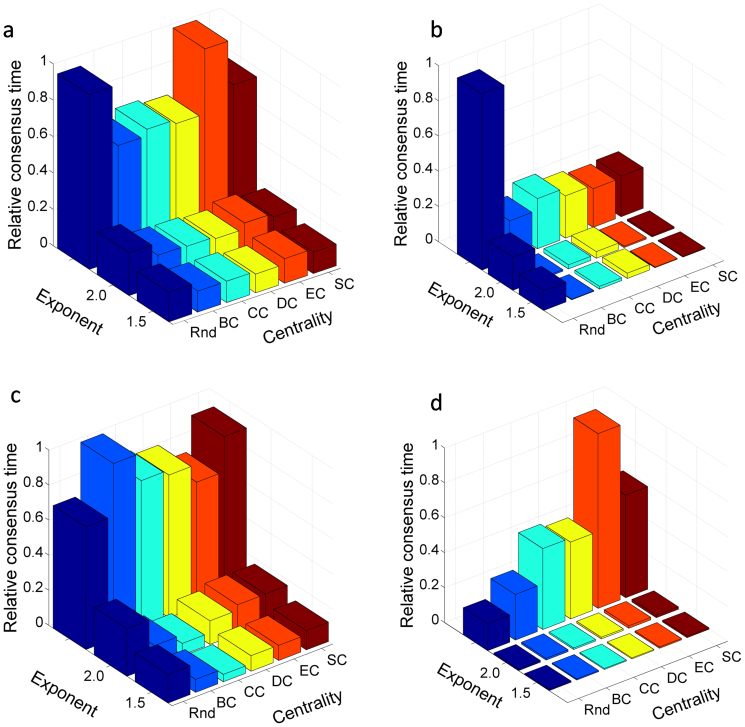
Random and centrality-based emergence of leaders. The emergence of leaders is analyzed according to randomness (Rnd), betweenness (BC), closeness (CC), degree (DC), eigenvector (EC), and subgraph (SC) centrality. The peer pressure is modeled by Δ*_d_* ~ *d^α^*, with *α* equal to −1.5 and −2.0. The third line corresponds to no peer pressure. (a) Communication network among workers in a sawmill. (b) Elite corporate directors. (c) Friendship network of injected drug users in Colorado Springs. (d) Random network having communities.

We now compare the hypotheses about the random emergence of good leaders—those who significantly reduce the time for reaching consensus in a network—to those in which leaders emerge from the most central individuals. Let us examine the emergence of leadership from five centrality criteria: degree, eigenvector, closeness, betweenness, and subgraph (see Supplementary Information equations (S1)–(S5)). In general, we observe that the leaders emerging from the most central individuals are better in leading the consensus than those emerging randomly. However, when there is certain level of PP over the actors, the situation changes dramatically (Fig. 1a, b). First, the time to reach consensus significantly decreases to less than 20% of the time needed when no PP exists. Second, a leader emerging randomly in the network could be as good as one emerging from the most central actors when PP exists in the system. Due to the recent results about the role of low-degree nodes in controlling complex networks^19^ we have also tested the role of PP over these potential drivers. Our results show again that good leaders emerge regardless of their centrality in the network when PP exists in the system (Supplementary Information). In other words, under the appropriate PP any individual in a social group could emerge as a good leader independently of her position in the network. This result adds a new dimension to the problem of network controllability^19^,^20^,^21^,^22^ by demonstrating that PP is a major driving force in determining how potential controllers can emerge in the network independently of their centrality (Supplementary Fig. S1) and — in contrast with previous results^19^,^23^,^24^ — of the degree distribution of the network (Supplementary Fig. S2).

In roughly half of the 15 social networks studied (Supplementary Information) we observe the following anomalous pattern. Leaders randomly emerging in the network are better in leading the consensus than some emerging from the most central individuals (see Fig. 1c). This situation appears when the network has the leaders distributed through diverse communities in the network. A *community* is a group of individuals who are more tightly connected among themselves than with the other actors in the network^25^. Actors in one of these communities reach consensus among themselves easily, but it is difficult to reach consensus between different communities. Most central actors in such networks are frequently located in a single community. When they emerge as leaders, they drive consensus only in their community but not in the global network. In contrast, when leaders emerge randomly, they more likely emerge simultaneously in different communities, a situation that favors global agreement in the network. Constructing a random network with communities as illustrated in Fig. 1d corroborates this hypothesis (Supplementary Tables S6 and S7). These results suggest the necessity of considering community leaders in social networks as effective mobilizers of actors throughout the network. We have observed that the leaders emerging on the basis of their community positions exhibit greater success in reaching consensus than those randomly emerging in the network. However, when appropriate PP exists, leaders who effectively reach consensus emerge regardless of their position in their communities.

**Figure 2:**
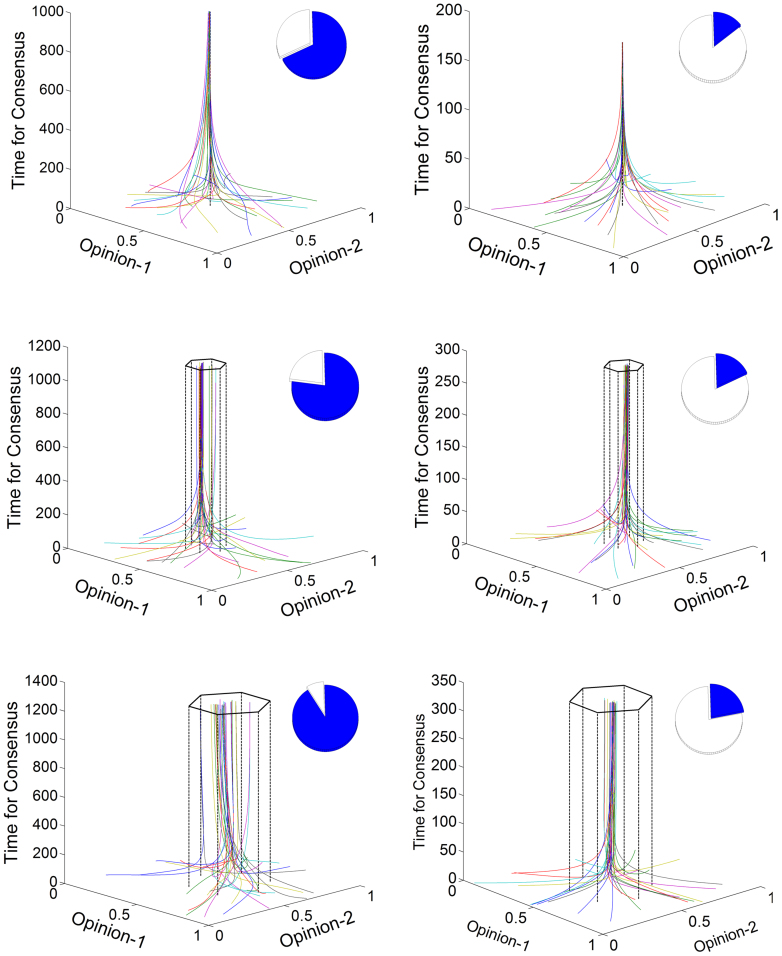
Leaders' cohesiveness and consensus. Analysis of the influence of leaders cohesiveness on the time to reach consensus in the communication network among workers in the sawmill without (left plots) and with (right plots) PP. The leaders' divergences used are: 0.0 (top), 0.1 (middle), and 0.2 (bottom). The time to reach consensus (in blue) relative to a total time of 1,500 units (Insets).

The leaders in a social group do not always exhibit a high level of cohesiveness. We posit that the leaders' capacity to lead the consensus in a network depends on their divergence of opinions. A cohesive group of leaders can more effectively lead the social group than leaders with larger divergences among their opinions. To model leader cohesiveness we introduce the divergence parameter ∇*_L_*, which is the circumradius of the regular polygon comprising all the leaders. ∇*_L_* = 0 indicates a very cohesive group of leaders. We now examine the influence of the leaders' cohesiveness on consensus. Figure 2 illustrates the results for the friendship network of workers in the sawmill with either no PP (left plots) or with PP modeled by Δ*_d_* ~ 1/*d*^2^ (right plots). The values of leader divergence range from 0.0 to 0.2. The lack of leader cohesiveness significantly increases the time to consensus when there is no PP. In fact, the time increases more than 33% when the divergence changes from 0.0 to 0.2 (it grows to 80.2% for ∇*_L_* = 0.5, see Supplementary Figs. S3 and S4 and Supplementary Tables S1, S3–S5). In addition, the cohesiveness of the group—measured by the standard deviation at consensus ∇*_G_*—is very poor for large values of ∇*_L_* (∇*_G_* = 154.6, 183.6, and 226.9 for ∇*_L_* = 0.0, 0.1, and 0.2, respectively), which indicates highly heterogeneous group opinions. However, when PP exists, the situation dramatically changes. First, the time to consensus does not increase as drastically with the decrease of leader cohesiveness. Second, group cohesiveness at the consensus is very high even for the lowest leader cohesiveness (∇*_G_* = 27.0, 35.4, and 33.0, for ∇*_L_* = 0.0, 0.1, and 0.2, respectively). In short, when PP is absent, leader cohesiveness plays a fundamental role in the time needed to reach consensus and in group cohesiveness at the consensus. When PP is present, the time needed to reach consensus and group cohesiveness are largely independent of the degree of divergence in the leaders' opinions, and the consensus is driven primarily by the influence of the nearest neighbors and PP.

### Diffusion of innovations and PP

Another area that has received great research attention is the diffusion of innovations^26^,^27^,^28^,^29^. The diffusion of innovations refers to the process through which new ideas and practices spread within and between social groups. Here we consider the hypothesis that PP plays a fundamental role in innovation adoption or rejection. To test our hypothesis, we study two datasets in which diffusion of innovations was followed for different periods of time (Supplementary Information). The first study analyzed the diffusion of a modern mathematic method among the primary and secondary schools in Allegheny County (Pennsylvania, USA). Results revealed that innovation diffused through the friendship network of the superintendents of the schools involved. The study was followed for a period of six years, 1958–1963. The second dataset represents the second phase of a longitudinal study about how Brazilian farmers adopted the use of hybrid seed corns, examining personal factors influencing farmers' innovative behavior in agriculture. We consider here the social network of friendship ties and the cumulative number of adopters of the new technology in three different communities of the Brazilian farmers study (Supplementary Fig. S5). The study was conducted over the course of 20 years and we consider only the individuals in the largest connected components of the networks.

**Figure 3:**
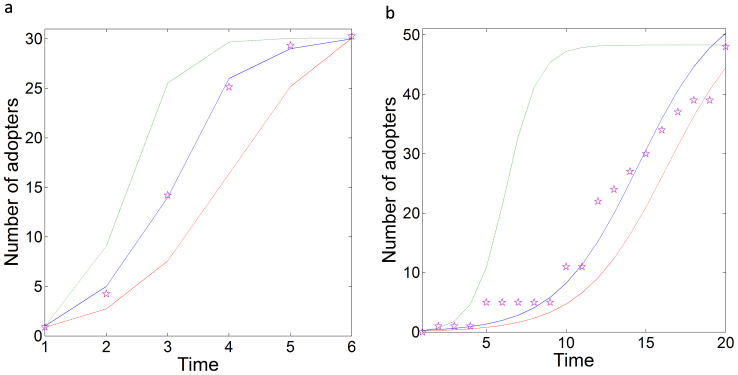
Diffusion of innovations under PP. (a) Adopters of a new mathematical method among US colleges in a period of 6 years. (b) Adopters of the use of hybrid seed corns among Brazilian farmers for a period of 20 years. Experimental values are given as stars and the simulation with no (broken red line), moderate (continuous blue line) and strong (dotted green line) PP are illustrated.

Figure 3 depicts the number of actors that adopted the respective innovations at different times. These values correspond to the number of adopters observed empirically in field studies. To simulate the process of innovation adoption, we study the consensus dynamics with equation (2), assuming Δ*_d_* ~ *d^α^*: no PP, moderate PP (−6.0 ≤ *α* ≤ −5.0), high PP (−4.0 ≤ *α* ≤ −3.0) (see Supplementary Information). The simulations follow perfect sigmoid curves, as Fig. 3 illustrates. Observe that when there is no PP effect, the diffusion curves predict slower rates of adoption than those empirically observed. For example, the empirical evidence demonstrates that 50% of schools adopted the new math method in roughly three years, whereas the simulation without PP predicts a period of four years of a total of six years. In the case of the Brazilian farmers, the empirical time for 50% of the farmers to adopt the innovation is roughly 12 years, whereas the simulation without PP predicts 16 years of a total of 20 years. When the model uses strong PP, the diffusion curves display very rapid adoption rates, which are far from the reality of the empirical evidence in both cases. However, using a moderate PP predicts very well the outputs of the empirical results in both studies. These PP values are found by a reverse engineering method, but the important message is that a certain PP level is necessary to describe the empirical evidence on the diffusion of innovations in social groups (see also Supplementary Information).

These results demonstrate that interpersonal communication alone cannot sufficiently explain the process of innovation adoption in a social group. The pressure exerted by the social group plays a fundamental role in shaping this important social phenomenon. Our model describes effectively PP's role in these and other important phenomena, consistent with our intuition and with the existing empirical evidence.

## Discussion

In this work, we have presented a methodology to address the previously unexplored influence of the combined action of direct and indirect peer pressure on social group dynamics. The developed model considers that the consensus dynamics is controlled not only by the agreement between directly connected peers, but also by the influence of those peers which are socially or culturally close to them. The results obtained with this generalized consensus model highlight the important role played by the indirect peer pressure on the processes of consensus, emergence of leadership and diffusion of innovations in social groups.

Consensus is known to be influenced by a small group of leaders who guides the behavior of the whole network^13^,^14^,^15^,^16^,^17^,^18^. The role of these drivers in the system controllability, and in particular their status or position in the complex network, has received great importance recently^19^,^20^,^21^,^22^,^23^,^24^. As expected the presence of these leaders reduces significantly the time for consensus in the network. In terms of controlling the system we show here that appropriate levels of indirect peer pressure allows that randomly emerging leaders could be as good as those occupying special positions or centrality in the network.

We also explore the role of two factors that have been previously ignored in the analysis of network controllability. The first is the role played by the presence of tightly connected groups or communities of nodes. The other is the cohesiveness of the leaders trying to drive the consensus of the whole network. In both cases we show here that if the level of indirect peer pressure is relatively weak, local leaders and leaders with strong cohesiveness are the best in controlling the network. However, as the indirect peer pressure increases the barriers imposed by the communities and leader cohesiveness vanish, and the networks are easily controlled even by leaders emerging from random positions.

Another area in which we have found that the indirect peer pressure plays a fundamental role is in the diffusion of innovations. In this case we show, with the help of real-world data about the diffusion of innovations in two different scenarios, that a moderate indirect peer pressure is needed in order to reproduce the rates of diffusion of these innovations independently of the social scenario in which they take place.

Our results not only offer a new perspective for the analysis of consensus in social groups, but also raise questions about the role of indirect peer pressure in the controllability of social networks. Future researches must explore how indirect peer pressure influences social activities in networks with very different topologies. Other models, apart from the consensus dynamics, can also be adapted to account for indirect peer pressure, opening new avenues in the analysis of these networked systems.

## Methods

### Consensus dynamics model

We consider a social group of *n* actors who will accomplish a certain goal or reach an agreement. Every actor in the group is represented by an element of the node set *V* = {1,…,*n*} of a network *G* = (*V*, *E*), in which links (edges) 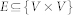 represent the relationships (friendship, any form of communication) among the actors. The set of neighbors of the actor *i* is denoted by 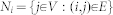. Let 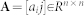 and 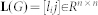 be the adjacency matrix and Laplacian matrix, respectively, associated with graph *G*. The Laplacian matrix is defined as **L** = **K** − **A**, where **K** is the diagonal matrix of node degrees of *G* and **A** is the adjacency matrix.

The information states of the actors evolve according to the single-integrator dynamics given by 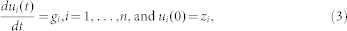
where 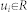 is the information state at time *t*, 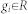 is the information control input, and 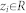 is the initial state of actor *i*, which is always considered to be selected at random. A continuous time consensus algorithm is given by 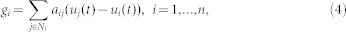
where *a_ij_* is the (*i*, *j*) entry of the adjacency matrix **A**. The information state of each actor is driven toward those of her neighbors. Equations (3) and (4) describe the collective dynamics of the social group and can be written in matrix form as 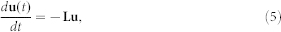
where **u** = [*u*_1_,…,*u_n_*]*^T^* is the vector of the states of the actors in the system. The consensus among the actors is achieved if, for all *u_i_*(*t*) and all *i*, *j* = 1,…,*n*, 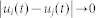 as *t* → ∞.

When the interaction among agents occurs at a discrete time, the information state is updated using a difference equation, and a discrete time consensus algorithm is then given by 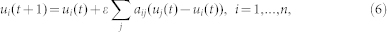
where *a_ij_* is as before and *ε* is the time step. The information state of each actor is updated as the weighted average of her current state and those of her neighbors. Equation (6) is written in matrix form as 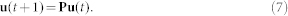
The matrix **p** is known as the Perron matrix, which is obtained as **P** = **I** − *ε***L**, for 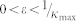, where κ_max_ is the maximum of the degrees of the nodes of *G*. The entries of the Perron matrix satisfy the property 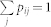 with *p_ij_* ≥ 0, µ*i*, *j*, and hence, it is a valid transition matrix^9^.

### Consensus with leaders–followers

We consider that there exist one or multiple leaders who guide the entire group to the consensus through the effect produced by the rest of the group, which follows them^30^. In a leaders–followers structure with a single leader, actors attempt to reach an agreement that is biased to the state of the leader, whereas in the case of multiple (stationary) leaders, all followers converge to the convex hull formed by the leaders' states.

An actor is called a stationary *leader* if her opinion is available for the other actors but is not modified during the process. Then, the set of all actors can be divided into two subgroups: leaders and followers. As a result, the vector of the states of all actors can also be divided into two parts: the states of leaders, *u_l_*, and the states of followers, *u_f_*.

For a system with multiple stationary leaders, all the nodes can be labeled such that the first *n_f_* represents the followers and the remaining *n_l_* represent the leaders. The total number of actors in the system is *n = n_f_ + n_l_*, such that the Laplacian matrix associated with the social network *G* is partitioned as 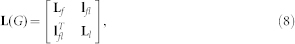
where 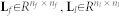, and 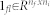.

Because the leaders are stationary, their dynamics are given by *u_i_*(*t*) = 0, *i* = *n_f_* + 1,…,*n*. Then, the dynamics of the system are expressed by 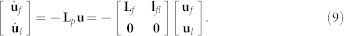
The discrete version of equation (9) is given by 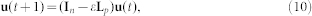
where 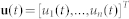, **I***_n_* is the identity matrix of size *n* × *n*, and **L***_p_* is the Laplacian matrix of network *G*, with each entry of the *j*th row equal to zero for *j* = *n_f_* + 1,…,*n*.

### Modeling peer pressure

The consensus dynamic modeling assumes that the actors only interact with their directly connected neighbors to cooperatively achieve an agreement in the system^31^. However, in many real-world situations, the actors are exposed not only to their closest contacts but also to individuals who are socio-culturally close to them despite not being directly connected. For instance, this situation appears in actors' attitudes toward copying others. The predisposition of an actor to copy a behavior depends not only on her friends' adoption of such behavior but also on other, socio-culturally close people having a positive predisposition to that behavior. For instance, adolescents adopt “binge drinking” not only by copying their mates but also by observing similar behavior among others of a similar age, education, and social class. Then, we argue that this socio-cultural distance can be captured in a model by considering the shortest path distance between two actors in their social group. The shortest path distance is the number of steps in the shortest path connecting the two actors. The influence that an actor receives/produces from/for others in her social network, i.e., peer pressure, decays as a function of this socio-cultural distance, which separates the two actors^32^.

Peer pressure can then be modeled by considering the generalized Laplacian matrix^33^. Consequently, the consensus dynamics model of equation (6) can be written as 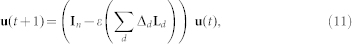
where 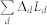 involves the *d*-Laplacian matrices and the coefficients Δ*_d_* indicate the strength of the interactions at distance *d* ≤ *d*_max_(*G*), with *d*_max_(*G*) being the maximum distance between two nodes or the diameter of graph *G*. The *d*-Laplacian matrix is defined as^33^

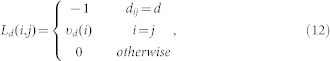
where the expression *υ_d_*(*i*) is the *d*-path degree of node *i* defined as the number of non-redundant shortest paths of length *d* having *i* as an endpoint.

The coefficients Δ*_d_* should account for the decay in peer pressure for the socio-cultural distance between the actors of Δ*_d_* ~ *f*(*d*)^−1^, where *f*(*d*) represents a function of distance *d*. In this study, we consider three different decay behaviors described by the following equations:

1. Power-law decay: Δ*_d_* = *d*^−*α*^,
2. Exponential decay: Δ*_d_* = *e*^−*βd*^, and
3. Social interactions: Δ*_d_* = *dδ^d^*^ − 1^,

where α, β, and δ are parameters to be adjusted to consider the different strengths of peer pressure.

## Author Contributions

E.V.E. collected data, performed research and analyzed data. E.E. designed and performed research, analyzed data and wrote the paper. Both authors discussed the results and commented on the manuscript.

## Supplementary Material

Supplementary Information Peer Pressure Shapes Consensus, Leadership, and Innovations in Social Groups
