# Supplementary material for: How Peer Pressure Shapes Consensus, Leadership, and Innovations in Social Groups
Source: Sci Rep. 2013 Oct 9;3:2905. doi: 10.1038/srep02905 (PMC3793217; doi:10.1038/srep02905)
Supplement: Supplementary Information — Peer Pressure Shapes Consensus, Leadership, and Innovations in Social Groups [file srep02905-s1.doc]

# Peer Pressure Shapes Consensus, Leadership, and Innovations in Social Groups

# Supplementary Information

Ernesto Estrada & Eusebio Vargas-Estrada

Department of Mathematics & Statistics, University of Strathclyde, Glasgow G1 1XH, UK

This file includes the following information:

Supplementary Methods and Discussion

Supplementary Dataset Summary

Supplementary Figures S1–S3

Supplementary Tables S1–S39

Supplementary References

Supplementary Methods and Discussion

**Emergence of leaders.** We consider two possible scenarios that describe the manner in which leaders emerge in a social group:

1. Emerging randomly from the social group, and
2. Emerging from the most central individuals in the social group.

The centrality of an actor in her social group can be considered in several ways. The concept of actor centrality is related to the question “Which are the most *important* or *central* nodes in a network?” We study five centrality measures defined in^25^ to consider potential leaders from among the actors in specific respective social groups. The centrality measures considered are as follows:

1. *Degree centrality* (DC): This measure is considered the simplest in a network defined as the number of edges connected to a node. It has been used assuming that nodes with connections to many other nodes might have more influence or access to information than those with few connections.

The degree of a node can be expressed in a matrix as

$\kappa_{i}=(\mathbf{A1})_{i}$, (S1)

where **1** is a column vector of ones and **A** is the adjacency matrix of the network.

1. *Eigenvector centrality* (EC): This measure appears as an extension of the degree of centrality. Eigenvector centrality is based on the question that not all neighbors are equivalent because, in some cases, the importance of a node is related to (and increased by) its neighbors, which may themselves be important. Thus, instead of giving only one point for each neighbor, this measure gives each node a score proportional to the sum of its neighbor’s scores.

The eigenvector centrality of node
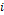
 is given by the
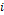
th entry of the principal eigenvector of the adjacency matrix

$\phi_{i}(i)=\left(\frac{1}{\lambda_{1}}\mathbf{A}\phi_{1}\right)_{i}$. (S2)

1. *Closeness centrality* (CC): This index measures the inverse of the mean distance from a node to other nodes and characterizes the nodes according to their distance to all other nodes in the network. The closeness is defined as

$CC(u)=\frac{n-1}{s(u)}$, (S3)

where $s(u)=\sum_{v\in V(G)} d(u,v)$is the distance sum of node *u*.

1. *Betweenness centrality* (BC): This concept measures the extent to which a node lies on paths between other nodes. The nodes with high betweenness centrality may have considerable influence within a network because of their control over information passing through them.

The index can be defined as

$BC=\sum_{i} \sum_{j} \frac{\rho(i,k,j)}{\rho(i,j)}, i\neq j\neq k$, (S4)

where $\rho(i,j)$ is the number of shortest paths from node *i* to *j*, and $\rho(i,k,j)$ is the number of these shortest paths that pass through node *k*.

1. *Subgraph centrality* (SC): This measure is based on the notion that the importance of a node can be characterized by considering its participation in all closed walks for which it is the starting point.

Subgraph centrality has been defined as

$EE(i)=\left(\sum_{l=0}^{\infty} \frac{\mathbf{A}^{l}}{l!}\right)_{ii}=\left(e^{\mathbf{A}}\right)_{ii}$. (S5)

Due to recent discoveries^19^ about the role of nodes with low degree as controllers or drivers in complex networks we have also studied the role of PP when the leaders emerge from nodes with high, medium and low degree. In the Supplementary Figure S1 we illustrate the results for two social networks which show that the effect of PP over the emergence of leaders is independent of the status of the nodes in their complex networks. In addition we display in Supplementary Figure S2 the cumulative degree distributions for all the social networks studied here. Our results resumed in Supplementary Tables S12-S39 show that the effect of PP is also independent of the degree distribution of the networks.

**Divergence of leaders’ opinions.** We consider that leaders’ opinions may differ from the group’s average opinion. We call this difference the divergence , which is represented by the circumradius of the regular polygon (for a two-dimensional case) that covers all opinions of the leaders (see Supplementary Fig. S3). If the concerned problem is multidimensional, that is, if we are considering more than two opinions in the system, then the divergence is the circumradius of a hypersphere that covers the opinion of all leaders.

We applied our model to two simple undirected random graphs and 15 real social networks described in the Dataset summary. We allowed for every actor to have two opinions that were considered independent from each other, enabling a two-dimensional decoupled consensus process. The initial states of the followers were randomly assigned for every process with values in the range (0, 1). We consider that only six leaders emerge, either randomly or from among the most central actors, who are determined according to their centralities values. Their initial positions were assigned to have divergences of 0.1 and 0.2 from the average consensus. The consensus dynamics stop when the difference between two consecutive measures of disagreement is less than or equal to 1e − 07. We simulate consensus processes with and without PP. The normalized values of every network are reported in Supplementary Tables S12–S39. Every value is the average of 50 realizations.

To test the effects of the divergence in the system dynamics, we used the social network Sawmill and simulated a consensus process that allowed for different PP intensities. We increased the value of divergence on leaders’ opinions from zero to a maximum of 0.5. We indicate the consensus times for all divergence values on Supplementary Tables S2–S5. For the case of randomly selected leaders, times increased along with divergence from 13.18% to 80.24% (see Supplementary Table S1).

We highlight that PP influenced the trajectories of followers’ opinions, precisely directing them toward the consensus space. At the consensus point, the final positions were more cohesive (Supplementary Fig. S4), indicating more homogeneous final opinions in the system.

**Networks with communities.** We discovered (see main text) that when leaders are spread among different tightly connected communities, “anomalous” patterns are observed in their emergence. This “anomaly” lies in the fact that leaders emerging randomly in the network are more efficient in reaching consensus than those emerging from among the most central actors. We tested this hypothesis by constructing random networks in which we control the number of communities as well as the connectivity within and among each community. These random networks with communities comprised simple, undirected random graphs with 500 nodes and 10 communities generated using the benchmarks for community detection in^34^ coded in the C programming language.

First, we selected 10 leaders randomly and by the global highest centralities and recorded the average time for consensus of 50 realizations. We allowed for the emergence of 10 leaders by the local (community-based) highest centrality, i.e., one leader in each community corresponding to the one with the highest centrality. When the leaders emerged from the global highest centralities, we observed the previously described anomalous behavior. This situation was modified when the leaders emerged from the community-based centrality. In this case, the leaders emerging from the most central local leaders were significantly better at reaching consensus than those emerging randomly (see Supplementary Tables S6 and S7).

**Diffusion of innovations.** The essence of the diffusion process is the information exchange by which one individual communicates a new idea to one or several others; thus, diffusion is a special type of communication concerned with the spread of messages perceived as new. In its most elementary form, the main elements in the diffusion process are^26^:

1. An innovation (message or information);
2. Communication channels, through which messages are conveyed from one individual to another;
3. Time of diffusion; and
4. The social system through which the process occurs—a set of interrelated units engaged in joint problem solving to accomplish a common goal. The members or units of a social system may be individuals, informal groups, organizations, and/or subsystems. All members cooperate at least to the extent of seeking to solve a common problem. This common objective binds the system.

Most innovations have a sigmoid-shaped (S-shaped) rate of adoption. The slope of the curve varies with every innovation. Certain new ideas diffuse relatively rapidly, and its S-curve is quite steep. Other innovations may have a slower rate of adoption, reflected by a more gradual S-curve. This behavior indicates the rate of adoption, i.e., the number of adopters of the new idea throughout time. The behavior of the adopters builds the form of the innovation process as follows^26^: Initially, few individuals adopt the innovation in each period; these are the innovators. Soon, the diffusion curve begins climbing as an increasing number of individuals adopt. Then, the trajectory of the rate of adoptions begins to level off, as fewer individuals remain who have not yet adopted. Finally, the S-curve reaches its asymptote and the diffusion process is complete.

Most individuals do not evaluate an innovation on the basis of scientific studies regarding its consequences; most people primarily depend on a subjective evaluation from individuals such as themselves who previously adopted the innovation, i.e., a dependency on the communicated experience of near peers.

In diffusion networks, certain individuals play different roles in a social system, and these roles affect diffusion. Certain members of the system function as opinion leaders: individuals who can influence others, and who are often identified and used to assure better diffusion of the information.

To analyze the impact of PP on the diffusion of innovations process, we used three networks from two empirical studies:

1. The network from the study Mathematical Method^35^: This innovation concerns the diffusion of a new mathematics method in the late 1950s. It was instigated by top mathematicians and sponsored by the U.S. National Science Foundation and the U.S. Department of Education. The diffusion process was successful because most schools adopted the new method. The example traces the diffusion of the modern mathematical method among school systems that combine elementary and secondary programs in Allegheny County (Pennsylvania, U.S.). All school superintendents who were in office for at least two years were interviewed.

Among other things, the superintendents were asked to indicate their friendship ties with other superintendents in the county through the following question: Among the chief school administrators in Allegheny County, who are your three best friends?

The researcher analyzed the friendship choices among superintendents who adopted the method and who were in office for at least one year before the first adoption, indicating that they could have adopted earlier. Unfortunately, the researcher did not include the friendship choices by superintendents who did not receive any choices themselves.

In our study, the network represents the friendship ties among the 30 superintendents who were part of the connected component, and the times for adoption represent the year in which the adopter chose the new mathematical method: 1-1958, 2-1959, 3-1960, 4-1961, 5-1962, and 6-1963.

1. Three networks from the study Brazilian Farmers^36^: “Diffusion and Adoption of Innovations in Rural Societies, 1952–1973,” was a longitudinal study on how Brazilian farmers (BF) adopted hybrid seed corns. The study was part of a broader, three-phase research project concerned with the spread of modern technology in Brazil, Nigeria, and India.

The data files reflect the second phase, which examined personal factors influencing farmers’ innovative agricultural behavior. Villages were selected from the total sample of Phase II villages. The groups of people were divided into different communities according to different variables, and the social networks of friends among the people in each community were retrieved.

The data used for our study includes the social network of friendship ties and the cumulative number of adopters of the new technology over 20 years among the individuals in the giant connected component for three different communities of the study, identified as communities 23, 70, and 71^37^.

We applied our consensus model to these networks, and the average consensus time of 50 realizations was divided into six intervals for the Mathematical Method and 20 intervals for the BF networks. We counted the number of actors or nodes corresponding to the average consensus at every time step by measuring the difference between a node’s position and the average consensus. When the absolute value of this difference was less than or equal to 0.04, we considered the node to be in agreement. This process was conducted with and without PP by considering a power-law decay.

The cumulative average of nodes in agreement at every interval is shown in Supplementary Tables S8–S11 for every network. In addition, the empirical cumulative number of adopters is indicated. We vary the values of parameter α to obtain behaviors that more effectively follow the empirical patterns. We divided these values into two classes: moderate PP ($-6.0\leq\alpha\leq-5.0$) and high PP ($-4.0\leq\alpha\leq-3.0$). Supplementary Figure S5 illustrates the curves of the diffusion processes that indicate that all results have sigmoid-like behavior that varies according to the PP in the system.

Supplementary Dataset Summary

| **Name** | **n** | **Description** ^25^ |
| --- | --- | --- |
| ER | 150 | Simple undirected random graph generated from the Erdös–Rényi model implemented in the toolbox CONTEST (Taylor and Higham, 2008). |
| BA | 150 | Simple undirected random graph generated from the preferential attachment model implemented in the toolbox CONTEST (Taylor and Higham, 2008). |
| Prison | 67 | Social network of prison inmates who chose “Which fellows on the tier are you closest friends with?” (MacRae, 1960) |
| Dolphins | 62 | An undirected social network of frequent associations between 62 dolphins (bottlenose) in a community living in New Zealand (Lusseau, 2003). |
| HS | 69 | Network of relations in a high school. The students choose the three members they wanted to have in a committee (Zeleny, 1950). |
| Zachary | 34 | Data collected by Wayne Zachary from the members of a university karate club, representing the presence or absence of ties among club members (Zachary, 1977). |
| Sawmill | 36 | A communication network within a small enterprise. All employees were asked to indicate the frequency with which they discussed work matters with each of their colleagues on a five-point scale ranging from less than once a week to several times a day (Michael and Massey, 1997; de Nooy *et al.*, 2005). |
| High Tech | 33 | A small high-tech computer firm that sells, installs, and maintains computer systems. The network contains the friendship ties among the employees (Krackhardt, 1999; de Nooy *et al.*, 2005). |
| Galesburg | 31 | From the Columbia University Drug Study. The diffusion of a new drug (gammanym) was investigated and the friendship ties among 31 physicians were coded (Coleman *et al.*, 1966; Knoke and Burt, 1983; de Nooy *et al.*, 2005). |
| Corporate | 1586 | American corporate elite, formed by directors of the 625 largest corporations, that reported the compositions of their boards, selected from the *Fortune* 1,000 in 1999 (Davis *et al*., 2003). |
| Drugs | 616 | Social network of injecting drug users (IDUs) who shared a needle in the last six months (Moody, 2001). |
| Colorado Springs | 324 | The risk network of persons with HIV during its early epidemic phase in Colorado Springs, U.S., selected through analysis of community-wide HIV/AIDS contact tracing records during 1985–1999 (Potterat *et al.*, 2002). |
| Math Method | 30 | This network concerns the diffusion of a new mathematics method in the 1950s. It traces the diffusion of the modern mathematical method among school systems that combine elementary and secondary programs in Allegheny County (Pennsylvania, U.S.) (de Nooy *et al.*, 2004). |
| Social3 | 32 | Network of social contacts among college students participating in a leadership course. The students choose the three members they wished to include in a committee (Zeleny, 1950). |
| BF23, BF70, BF71 | 40, 48, 49 | Networks of friendship ties from the communities identified as 23, 70, and 71 from the Brazilian Farmers longitudinal study on the adoption of a new corn seed (Valente, 2012; Herzog *et al.*, 1968). |

**a**

**b**

**Supplementary Figure S1 | Influence of PP on emergence of leaders by selecting them among the nodes with high, medium or low degree centrality.** The HighTech (a) and the network of social dates (social3) (b).

|  |  |
| --- | --- |
|  |  |
|  |  |
|  |  |
|  |  |
|  |  |
|  |  |
|  |  |

**Supplementary Figure S2 | Cumulative degree distribution of the 15 social networks studied.** BF23, BF70, BF71, ColoSpg, Corporate, Dolphins, Drugs, Galesburg, HighTech, HS, MathMethod, Prison, SawMill, Social3, Zackary.

**Supplementary Figure S3 | Leaders with divergence.** Distribution of a leader’s positions (blue points) around the average consensus value of the system (red point), which represents the centroid of the convex hull spanned by the leaders.

|  |  |
| --- | --- |
|  |  |
|  |  |
|  |  |

**Supplementary Figure S4 |** **Consensus for the Sawmill network.** Consensus with divergences (rows from top to bottom): 0.0, 0.1, 0.2, and 0.5, without PP (left column) and with PP (right column). The continuous lines indicate the states of the followers and the discontinuous lines indicate the states of the leaders during the consensus process.

|  |  |
| --- | --- |
|  |  |

**Supplementary Figure S5 | Diffusion curves for empirical social networks.** The Mathematical Method (top left) and Brazilian Farmers Communities 23 (top right), 70 (bottom left), and 71(bottom right). The stars represent the empirical values of the cumulative number of adopters. The red lines represent diffusion without PP, the blue lines represent the diffusion process with moderate PP, and the green lines represent diffusion with strong PP.

Supplementary Table S1

Comparison of time for consensus for the Sawmill network with different values of divergence and the effect on time to reach consensus.

| **Divergence** | **Average Time for Consensus** | **% Increase in Time for Consensus** |
| --- | --- | --- |
| 0 | 1,026.80 | - |
| 0.1 | 1,162.18 | 13.18 |
| 0.2 | 1,372.40 | 33.65 |
| 0.5 | 1,850.72 | 80.24 |

Supplementary Table S2

Normalized consensus time for the Sawmill network with no divergence in leaders’ positions with respect to the system’s average consensus value. All leaders’ initial states were equal to the average consensus, i.e., the average of followers’ initial states.

|  | **Random Emergence** | | | | | | | |
| --- | --- | --- | --- | --- | --- | --- | --- | --- |
|  | **No PP** | **PL-decay** | | **Exp-decay** | | **Social** | | |
|  |  | α=-2 | α=-1.5 | β=-2 | β=-1.5 | δ=0.1 | δ=0.25 | δ=0.5 |
|  | 1.00 | 0.22 | 0.15 | 0.88 | 0.72 | 0.49 | 0.20 | 0.06 |
|  | **Leader-Centrality Emergence** | | | | | | | |
|  | **No PP** | **PL-decay** | | **Exp-decay** | | **Social** | | |
| **Centrality** |  | α=-2 | α=-1.5 | β=-2 | β=-1.5 | δ=0.1 | δ=0.25 | δ=0.5 |
| BC | 0.80 | 0.21 | 0.14 | 0.73 | 0.62 | 0.43 | 0.19 | 0.05 |
| CC | 0.84 | 0.21 | 0.14 | 0.77 | 0.65 | 0.45 | 0.19 | 0.05 |
| DC | 0.83 | 0.21 | 0.14 | 0.76 | 0.64 | 0.45 | 0.19 | 0.06 |
| EC | 0.96 | 0.23 | 0.15 | 0.87 | 0.73 | 0.50 | 0.20 | 0.06 |
| SC | 0.89 | 0.23 | 0.14 | 0.85 | 0.69 | 0.46 | 0.20 | 0.06 |

Supplementary Table S3

Normalized consensus time for the Sawmill network with divergence of 0.1 in leaders’ positions with respect to the system’s average consensus value, i.e., the length of the circumradius of the regular polygon spanned by the leaders is equal to 0.1.

|  | **Random Emergence** | | | | | | | |
| --- | --- | --- | --- | --- | --- | --- | --- | --- |
|  | **No PP** | **PL-decay** | | **Exp-decay** | | **Social** | | |
|  |  | α=-2 | α=-1.5 | β=-2 | β=-1.5 | δ=0.1 | δ=0.25 | δ=0.5 |
|  | 1.00 | 0.24 | 0.17 | 0.88 | 0.68 | 0.45 | 0.20 | 0.07 |
|  | **Leader-Centrality Emergence** | | | | | | | |
|  | **No PP** | **PL-decay** | | **Exp-decay** | | **Social** | | |
| **Centrality** |  | α=-2 | α=-1.5 | β=-2 | β=-1.5 | δ=0.1 | δ=0.25 | δ=0.5 |
| BC | 0.72 | 0.19 | 0.13 | 0.65 | 0.54 | 0.38 | 0.17 | 0.05 |
| CC | 0.75 | 0.19 | 0.12 | 0.70 | 0.56 | 0.40 | 0.17 | 0.05 |
| DC | 0.74 | 0.19 | 0.12 | 0.71 | 0.59 | 0.40 | 0.17 | 0.05 |
| EC | 0.99 | 0.22 | 0.15 | 0.92 | 0.68 | 0.45 | 0.18 | 0.05 |
| SC | 0.82 | 0.20 | 0.13 | 0.76 | 0.62 | 0.41 | 0.18 | 0.05 |

Supplementary Table S4

Normalized consensus time for the Sawmill network with divergence of 0.2 in leaders’ positions with respect to the system’s average consensus value, i.e., the length of the circumradius of the regular polygon spanned by the leaders is equal to 0.2.

|  | **Random Emergence** | | | | | | | |
| --- | --- | --- | --- | --- | --- | --- | --- | --- |
|  | **No PP** | **PL-decay** | | **Exp-decay** | | **Social** | | |
|  |  | α=-2 | α=-1.5 | β=-2 | β=-1.5 | δ=0.1 | δ=0.25 | δ=0.5 |
|  | 0.96 | 0.23 | 0.16 | 0.87 | 0.66 | 0.43 | 0.19 | 0.06 |
|  | **Leader-Centrality Emergence** | | | | | | | |
|  | **No PP** | **PL-decay** | | **Exp-decay** | | **Social** | | |
| **Centrality** |  | α=-2 | α=-1.5 | β=-2 | β=-1.5 | δ=0.1 | δ=0.25 | δ=0.5 |
| BC | 0.63 | 0.17 | 0.12 | 0.59 | 0.46 | 0.33 | 0.14 | 0.05 |
| CC | 0.66 | 0.16 | 0.12 | 0.58 | 0.49 | 0.33 | 0.14 | 0.04 |
| DC | 0.64 | 0.15 | 0.10 | 0.60 | 0.48 | 0.33 | 0.14 | 0.04 |
| EC | 1.00 | 0.19 | 0.13 | 0.85 | 0.63 | 0.37 | 0.15 | 0.05 |
| SC | 0.75 | 0.17 | 0.12 | 0.65 | 0.51 | 0.35 | 0.14 | 0.04 |

Supplementary Table S5

Normalized consensus time for the Sawmill network with divergence of 0.5 in leaders’ positions with respect to the system’s average consensus value, i.e., the length of the circumradius of the regular polygon spanned by the leaders is equal to 0.5.

|  | **Random Emergence** | | | | | | | |
| --- | --- | --- | --- | --- | --- | --- | --- | --- |
|  | **No PP** | **PL-decay** | | **Exp-decay** | | **Social** | | |
|  |  | α=-2 | α=-1.5 | β=-2 | β=-1.5 | δ=0.1 | δ=0.25 | δ=0.5 |
|  | 1.00 | 0.23 | 0.16 | 0.87 | 0.67 | 0.40 | 0.17 | 0.06 |
|  | **Leader-Centrality Emergence** | | | | | | | |
|  | **No PP** | **PL-decay** | | **Exp-decay** | | **Social** | | |
| **Centrality** |  | α=-2 | α=-1.5 | β=-2 | β=-1.5 | δ=0.1 | δ=0.25 | δ=0.5 |
| BC | 0.59 | 0.15 | 0.11 | 0.53 | 0.43 | 0.27 | 0.11 | 0.04 |
| CC | 0.60 | 0.14 | 0.11 | 0.52 | 0.41 | 0.27 | 0.11 | 0.04 |
| DC | 0.60 | 0.12 | 0.08 | 0.54 | 0.43 | 0.27 | 0.10 | 0.03 |
| EC | 0.99 | 0.17 | 0.12 | 0.83 | 0.59 | 0.35 | 0.13 | 0.04 |
| SC | 0.72 | 0.14 | 0.10 | 0.60 | 0.47 | 0.29 | 0.11 | 0.04 |

Supplementary Table S6

Normalized consensus times for a random graph with 10 communities (10 leaders) with and without PP. Leaders emerged according to their global centrality values.

|  | **Random Emergence** | | | | | | | |
| --- | --- | --- | --- | --- | --- | --- | --- | --- |
|  | **No PP** | **PL-decay** | | **Exp-decay** | | **Social** | | |
|  |  | α=-2 | α=-1.5 | β=-2 | β=-1.5 | δ=0.1 | δ=0.25 | δ=0.5 |
|  | 0.15 | 0.01 | 0.01 | 0.12 | 0.06 | 0.04 | 0.01 | 0.003 |
|  | **Leader-Centrality Emergence** | | | | | | | |
|  | **No PP** | **PL-decay** | | **Exp-decay** | | **Social** | | |
| **Centrality** |  | α=-2 | α=-1.5 | β=-2 | β=-1.5 | δ=0.1 | δ=0.25 | δ=0.5 |
| BC | 0.26 | 0.01 | 0.01 | 0.18 | 0.09 | 0.06 | 0.01 | 0.003 |
| CC | 0.46 | 0.01 | 0.01 | 0.32 | 0.16 | 0.10 | 0.02 | 0.002 |
| DC | 0.44 | 0.01 | 0.01 | 0.32 | 0.16 | 0.10 | 0.02 | 0.003 |
| EC | 1.00 | 0.01 | 0.01 | 0.62 | 0.27 | 0.16 | 0.03 | 0.003 |
| SC | 0.59 | 0.01 | 0.01 | 0.34 | 0.15 | 0.09 | 0.02 | 0.004 |

Supplementary Table S7

Normalized consensus times for a graph with 10 communities (10 leaders) with and without PP. Leaders emerged according to their centrality values by community.

|  | **Random Emergence** | | | | | | | |
| --- | --- | --- | --- | --- | --- | --- | --- | --- |
|  | **No PP** | **PL-decay** | | **Exp-decay** | | **Social** | | |
|  |  | α=-2 | α=-1.5 | β=-2 | β=-1.5 | δ=0.1 | δ=0.25 | δ=0.5 |
|  | 1.00 | 0.06 | 0.04 | 0.77 | 0.41 | 0.27 | 0.08 | 0.02 |
|  | **Leader-Centrality Emergence** | | | | | | | |
|  | **No PP** | **PL-decay** | | **Exp-decay** | | **Social** | | |
| **Centrality** |  | α=2 | α=1.5 | β=2 | β=1.5 | δ=0.1 | δ=0.25 | δ=0.5 |
| BC | 0.54 | 0.07 | 0.04 | 0.42 | 0.28 | 0.17 | 0.06 | 0.02 |
| CC | 0.40 | 0.07 | 0.04 | 0.31 | 0.22 | 0.14 | 0.06 | 0.02 |
| DC | 0.30 | 0.07 | 0.05 | 0.26 | 0.20 | 0.13 | 0.07 | 0.02 |
| EC | 0.43 | 0.07 | 0.04 | 0.34 | 0.25 | 0.15 | 0.06 | 0.02 |
| SC | 0.29 | 0.07 | 0.04 | 0.25 | 0.19 | 0.12 | 0.06 | 0.02 |

Supplementary Table S8

Cumulative average nodes in agreement for the Math Method network with and without PP and empirical values.

| **Periods** | **Adopters  (Empirical)** | **Avg. Adopters (Simulation)** | | |
| --- | --- | --- | --- | --- |
|  |  | *No LR* | *α=-4* | *α=-5* |
| 1 | 1 | 1.1 | 1.3 | 1.1 |
| 2 | 5 | 2.7 | 9.0 | 4.7 |
| 3 | 14 | 7.7 | 25.6 | 13.7 |
| 4 | 26 | 23.6 | 29.8 | 27.8 |
| 5 | 29 | 28.3 | 30 | 29.4 |
| 6 | 30 | 30 | 30 | 30 |

Supplementary Table S9

Cumulative average nodes in agreement for the Brazilian Farmers, Community 23 network with and without PP and empirical values.

| **Periods** | **Adopters  (Empirical)** | **Avg. Adopters (Simulation)** | | |
| --- | --- | --- | --- | --- |
|  |  | *No PP* | *α=-4* | *α=-5.9* |
| 1 | 1 | 0.8 | 1.0 | 0.8 |
| 2 | 1 | 1.3 | 1.8 | 1.2 |
| 3 | 1 | 1.2 | 2.0 | 1.1 |
| 4 | 1 | 1.5 | 3.0 | 1.5 |
| 5 | 3 | 1.8 | 7.2 | 1.7 |
| 6 | 3 | 2.0 | 15.8 | 2.2 |
| 7 | 3 | 2.3 | 23.4 | 2.4 |
| 8 | 4 | 2.5 | 26.0 | 4.1 |
| 9 | 4 | 4.0 | 32.3 | 9.0 |
| 10 | 6 | 5.6 | 35.9 | 10.9 |
| 11 | 6 | 7.2 | 37.2 | 11.5 |
| 12 | 11 | 8.9 | 38.5 | 12.5 |
| 13 | 13 | 9.5 | 38.9 | 13.5 |
| 14 | 15 | 10.3 | 38.9 | 15.8 |
| 15 | 19 | 12.3 | 38.9 | 17.3 |
| 16 | 23 | 13.4 | 38.9 | 19.8 |
| 17 | 33 | 19.0 | 38.9 | 24.6 |
| 18 | 33 | 24.9 | 38.9 | 26.9 |
| 19 | 37 | 26.4 | 38.9 | 30.4 |
| 20 | 38 | 27.1 | 38.9 | 32.9 |

Supplementary Table S10

Cumulative average nodes in agreement for the Brazilian Farmers, Community 70 network with and without PP and empirical values.

| **Periods** | **Adopters  (Empirical)** | **Avg. Adopters (Simulation)** | | |
| --- | --- | --- | --- | --- |
|  |  | *No PP* | *α=-4* | *α =-5.6* |
| 1 | 0 | 0.9 | 0.8 | 0.7 |
| 2 | 0 | 1.7 | 3.2 | 1.8 |
| 3 | 0 | 1.8 | 9.5 | 3.0 |
| 4 | 0 | 3.3 | 14.3 | 4.5 |
| 5 | 14 | 4.9 | 23.4 | 5.6 |
| 6 | 14 | 4.7 | 36.2 | 8.6 |
| 7 | 15 | 5.8 | 45.4 | 13.7 |
| 8 | 18 | 6.4 | 47.7 | 16.5 |
| 9 | 20 | 6.6 | 47.8 | 18.9 |
| 10 | 28 | 8.3 | 47.8 | 21.0 |
| 11 | 29 | 10.2 | 47.8 | 23.5 |
| 12 | 31 | 11.8 | 47.8 | 25.9 |
| 13 | 31 | 14.0 | 47.8 | 28.4 |
| 14 | 36 | 16.5 | 47.8 | 30.7 |
| 15 | 38 | 19.1 | 47.8 | 33.1 |
| 16 | 42 | 22.7 | 47.8 | 37.8 |
| 17 | 44 | 27.3 | 47.8 | 42.1 |
| 18 | 44 | 31.7 | 47.8 | 45.6 |
| 19 | 45 | 35.4 | 47.8 | 47.4 |
| 20 | 46 | 39.0 | 47.8 | 47.5 |

Supplementary Table S11

Cumulative average nodes in agreement for the Brazilian Farmers, Community 71 network with and without PP and empirical values.

| **Periods** | **Adopters  (Empirical)** | **Avg. Adopters (Simulation)** | | |
| --- | --- | --- | --- | --- |
|  |  | *No PP* | *α=-4* | *α=-6.6* |
| 1 | 0 | 0.5 | 0.7 | 0.7 |
| 2 | 1 | 0.9 | 1.9 | 1.1 |
| 3 | 1 | 1.2 | 4.0 | 1.4 |
| 4 | 1 | 1.1 | 7.3 | 1.6 |
| 5 | 5 | 1.0 | 11.8 | 3.2 |
| 6 | 5 | 1.2 | 21.6 | 5.5 |
| 7 | 5 | 2.1 | 32.2 | 6.4 |
| 8 | 5 | 2.9 | 37.3 | 6.6 |
| 9 | 5 | 3.6 | 46.0 | 7.0 |
| 10 | 11 | 4.6 | 47.8 | 7.9 |
| 11 | 11 | 6.1 | 48.4 | 9.6 |
| 12 | 22 | 8.5 | 48.4 | 12.7 |
| 13 | 24 | 11.7 | 48.4 | 17.3 |
| 14 | 27 | 16.4 | 48.4 | 23.8 |
| 15 | 30 | 21.9 | 48.4 | 31.5 |
| 16 | 34 | 26.6 | 48.4 | 38.2 |
| 17 | 37 | 30.8 | 48.4 | 43.1 |
| 18 | 39 | 36.2 | 48.4 | 45.9 |
| 19 | 39 | 40.8 | 48.4 | 47.4 |
| 20 | 48 | 44.5 | 48.4 | 47.6 |

Supplementary Table S12

Normalized consensus times for the BA random graph with divergence of 0.1.

|  | **Random Emergence** | | | | | | | |
| --- | --- | --- | --- | --- | --- | --- | --- | --- |
|  | **No PP** | **PL-decay** | | **Exp-decay** | | **Social** | | |
|  |  | α=-2 | α=-1.5 | β=-2 | β=-1.5 | δ=0.1 | δ=0.25 | δ=0.5 |
|  | 1.00 | 0.44 | 0.31 | 0.75 | 0.67 | 0.84 | 0.38 | 0.13 |
|  | **Leader-Centrality Emergence** | | | | | | | |
|  | **No PP** | **PL-decay** | | **Exp-decay** | | **Social** | | |
| **Centrality** |  | α=-2 | α=-1.5 | β=-2 | β=-1.5 | δ=0.1 | δ=0.25 | δ=0.5 |
| BC | 0.89 | 0.33 | 0.26 | 0.73 | 0.61 | 0.41 | 0.23 | 0.09 |
| CC | 0.91 | 0.34 | 0.27 | 0.78 | 0.65 | 0.42 | 0.22 | 0.08 |
| DC | 0.89 | 0.33 | 0.26 | 0.75 | 0.62 | 0.41 | 0.23 | 0.09 |
| EC | 0.93 | 0.34 | 0.27 | 0.77 | 0.64 | 0.42 | 0.22 | 0.08 |
| SC | 0.90 | 0.34 | 0.27 | 0.76 | 0.63 | 0.41 | 0.22 | 0.08 |

Supplementary Table S13

Normalized consensus times for the ER random graph with divergence of 0.1.

|  | **Random Emergence** | | | | | | | |
| --- | --- | --- | --- | --- | --- | --- | --- | --- |
|  | **No PP** | **PL-decay** | | **Exp-decay** | | **Social** | | |
|  |  | α=-2 | α=-1.5 | β=-2 | β=-1.5 | δ=0.1 | δ=0.25 | δ=0.5 |
|  | 1.00 | 0.34 | 0.23 | 0.90 | 0.75 | 0.61 | 0.31 | 0.10 |
|  | **Leader-Centrality Emergence** | | | | | | | |
|  | **No PP** | **PL-decay** | | **Exp-decay** | | **Social** | | |
| **Centrality** |  | α=-2 | α=-1.5 | β=-2 | β=-1.5 | δ=0.1 | δ=0.25 | δ=0.5 |
| BC | 0.81 | 0.10 | 0.06 | 0.67 | 0.47 | 0.28 | 0.10 | 0.05 |
| CC | 0.81 | 0.10 | 0.06 | 0.67 | 0.46 | 0.27 | 0.09 | 0.02 |
| DC | 0.81 | 0.10 | 0.06 | 0.69 | 0.48 | 0.28 | 0.10 | 0.03 |
| EC | 0.82 | 0.13 | 0.08 | 0.69 | 0.48 | 0.30 | 0.11 | 0.03 |
| SC | 0.82 | 0.13 | 0.08 | 0.68 | 0.48 | 0.31 | 0.11 | 0.02 |

Supplementary Table S14

Normalized consensus times for the Corporate network with divergence of 0.1.

|  | **Random Emergence** | | | | | | | |
| --- | --- | --- | --- | --- | --- | --- | --- | --- |
|  | **No PP** | **PL-decay** | | **Exp-decay** | | **Social** | | |
|  |  | α=-2 | α=-1.5 | β=-2 | β=-1.5 | δ=0.1 | δ=0.25 | δ=0.5 |
|  | 1.00 | 0.36 | 0.22 | 0.96 | 0.92 | 0.94 | 0.37 | 0.11 |
|  | **Leader-Centrality Emergence** | | | | | | | |
|  | **No PP** | **PL-decay** | | **Exp-decay** | | **Social** | | |
| **Centrality** |  | α=-2 | α=-1.5 | β=-2 | β=-1.5 | δ=0.1 | δ=0.25 | δ=0.5 |
| BC | 0.67 | 0.02 | 0.01 | 0.52 | 0.35 | 0.27 | 0.08 | 0.01 |
| CC | 0.70 | 0.03 | 0.01 | 0.54 | 0.35 | 0.28 | 0.09 | 0.01 |
| DC | 0.69 | 0.05 | 0.04 | 0.52 | 0.36 | 0.29 | 0.10 | 0.02 |
| EC | 0.67 | 0.02 | 0.01 | 0.52 | 0.36 | 0.27 | 0.08 | 0.01 |
| SC | 0.68 | 0.02 | 0.01 | 0.51 | 0.35 | 0.27 | 0.08 | 0.01 |

Supplementary Table S15

Normalized consensus times for the Drugs network with divergence of 0.1.

|  | **Random Emergence** | | | | | | | |
| --- | --- | --- | --- | --- | --- | --- | --- | --- |
|  | **No PP** | **PL-decay** | | **Exp-decay** | | **Social** | | |
|  |  | α=-2 | α=-1.5 | β=-2 | β=-1.5 | δ=0.1 | δ=0.25 | δ=0.5 |
|  | 1.00 | 0.17 | 0.12 | 0.89 | 0.64 | 0.53 | 0.24 | 0.08 |
|  | **Leader-Centrality Emergence** | | | | | | | |
|  | **No PP** | **PL-decay** | | **Exp-decay** | | **Social** | | |
| **Centrality** |  | α=-2 | α=-1.5 | β=-2 | β=-1.5 | δ=0.1 | δ=0.25 | δ=0.5 |
| BC | 0.97 | 0.07 | 0.04 | 0.79 | 0.51 | 0.40 | 0.12 | 0.02 |
| CC | 0.79 | 0.08 | 0.05 | 0.73 | 0.50 | 0.40 | 0.12 | 0.02 |
| DC | 0.77 | 0.04 | 0.06 | 0.66 | 0.45 | 0.36 | 0.13 | 0.06 |
| EC | 0.80 | 0.11 | 0.08 | 0.71 | 0.53 | 0.43 | 0.13 | 0.04 |
| SC | 0.80 | 0.11 | 0.08 | 0.72 | 0.50 | 0.42 | 0.13 | 0.04 |

Supplementary Table S16

Normalized consensus times for the Prison network with divergence of 0.1.

|  | **Random Emergence** | | | | | | | |
| --- | --- | --- | --- | --- | --- | --- | --- | --- |
|  | **No PP** | **PL-decay** | | **Exp-decay** | | **Social** | | |
|  |  | α=-2 | α=-1.5 | β=-2 | β=-1.5 | δ=0.1 | δ=0.25 | δ=0.5 |
|  | 1.00 | 0.26 | 0.19 | 0.93 | 0.70 | 0.49 | 0.23 | 0.08 |
|  | **Leader-Centrality Emergence** | | | | | | | |
|  | **No PP** | **PL-decay** | | **Exp-decay** | | **Social** | | |
| **Centrality** |  | α=-2 | α=-1.5 | β=-2 | β=-1.5 | δ=0.1 | δ=0.25 | δ=0.5 |
| BC | 0.93 | 0.17 | 0.11 | 0.85 | 0.68 | 0.45 | 0.17 | 0.04 |
| CC | 0.97 | 0.17 | 0.11 | 0.86 | 0.66 | 0.45 | 0.17 | 0.04 |
| DC | 0.95 | 0.17 | 0.10 | 0.85 | 0.65 | 0.44 | 0.17 | 0.04 |
| EC | 0.98 | 0.17 | 0.11 | 0.84 | 0.68 | 0.44 | 0.17 | 0.04 |
| SC | 0.94 | 0.17 | 0.10 | 0.85 | 0.65 | 0.43 | 0.17 | 0.04 |

Supplementary Table S17

Normalized consensus times for the Zachary network with divergence of 0.1.

|  | **Random Emergence** | | | | | | | |
| --- | --- | --- | --- | --- | --- | --- | --- | --- |
|  | **No PP** | **PL-decay** | | **Exp-decay** | | **Social** | | |
|  |  | α=-2 | α=-1.5 | β=-2 | β=-1.5 | δ=0.1 | δ=0.25 | δ=0.5 |
|  | 1.00 | 0.27 | 0.19 | 0.94 | 0.67 | 0.39 | 0.19 | 0.08 |
|  | **Leader-Centrality Emergence** | | | | | | | |
|  | **No PP** | **PL-decay** | | **Exp-decay** | | **Social** | | |
| **Centrality** |  | α=-2 | α=-1.5 | β=-2 | β=-1.5 | δ=0.1 | δ=0.25 | δ=0.5 |
| BC | 0.73 | 0.22 | 0.17 | 0.63 | 0.51 | 0.32 | 0.16 | 0.06 |
| CC | 0.75 | 0.22 | 0.16 | 0.66 | 0.50 | 0.32 | 0.15 | 0.06 |
| DC | 0.76 | 0.22 | 0.17 | 0.64 | 0.51 | 0.32 | 0.16 | 0.06 |
| EC | 0.75 | 0.21 | 0.17 | 0.64 | 0.50 | 0.33 | 0.16 | 0.05 |
| SC | 0.76 | 0.22 | 0.17 | 0.65 | 0.49 | 0.31 | 0.16 | 0.06 |

Supplementary Table S18

Normalized consensus times for the Colorado Springs network with divergence of 0.1.

|  | **Random Emergence** | | | | | | | |
| --- | --- | --- | --- | --- | --- | --- | --- | --- |
|  | **No PP** | **PL-decay** | | **Exp-decay** | | **Social** | | |
|  |  | α=-2 | α=-1.5 | β=-2 | β=-1.5 | δ=0.1 | δ=0.25 | δ=0.5 |
|  | 0.83 | 0.22 | 0.15 | 0.76 | 0.62 | 0.56 | 0.37 | 0.13 |
|  | **Leader-Centrality Emergence** | | | | | | | |
|  | **No PP** | **PL-decay** | | **Exp-decay** | | **Social** | | |
| **Centrality** |  | α=-2 | α=-1.5 | β=-2 | β=-1.5 | δ=0.1 | δ=0.25 | δ=0.5 |
| BC | 1.00 | 0.11 | 0.07 | 0.87 | 0.69 | 0.53 | 0.25 | 0.04 |
| CC | 0.90 | 0.07 | 0.03 | 0.82 | 0.53 | 0.27 | 0.27 | 0.04 |
| DC | 0.89 | 0.13 | 0.09 | 0.82 | 0.65 | 0.50 | 0.24 | 0.06 |
| EC | 0.85 | 0.13 | 0.06 | 0.75 | 0.62 | 0.47 | 0.22 | 0.04 |
| SC | 0.95 | 0.17 | 0.12 | 0.86 | 0.69 | 0.54 | 0.28 | 0.07 |

Supplementary Table S19

Normalized consensus times for the Dolphins network with divergence of 0.1.

|  | **Random Emergence** | | | | | | | |
| --- | --- | --- | --- | --- | --- | --- | --- | --- |
|  | **No PP** | **PL-decay** | | **Exp-decay** | | **Social** | | |
|  |  | α=-2 | α=-1.5 | β=-2 | β=-1.5 | δ=0.1 | δ=0.25 | δ=0.5 |
|  | 0.82 | 0.25 | 0.16 | 0.76 | 0.61 | 0.38 | 0.20 | 0.08 |
|  | **Leader-Centrality Emergence** | | | | | | | |
|  | **No PP** | **PL-decay** | | **Exp-decay** | | **Social** | | |
| **Centrality** |  | α=-2 | α=-1.5 | β=-2 | β=-1.5 | δ=0.1 | δ=0.25 | δ=0.5 |
| BC | 0.77 | 0.17 | 0.11 | 0.70 | 0.56 | 0.37 | 0.15 | 0.06 |
| CC | 0.92 | 0.17 | 0.12 | 0.65 | 0.64 | 0.43 | 0.18 | 0.05 |
| DC | 0.72 | 0.18 | 0.12 | 0.64 | 0.57 | 0.39 | 0.17 | 0.06 |
| EC | 0.96 | 0.21 | 0.14 | 0.99 | 0.79 | 0.52 | 0.21 | 0.06 |
| SC | 1.00 | 0.22 | 0.14 | 0.97 | 0.77 | 0.54 | 0.21 | 0.06 |

Supplementary Table S20

Normalized consensus times for the Galesburg network with divergence of 0.1.

|  | **Random Emergence** | | | | | | | |
| --- | --- | --- | --- | --- | --- | --- | --- | --- |
|  | **No PP** | **PL-decay** | | **Exp-decay** | | **Social** | | |
|  |  | α=-2 | α=-1.5 | β=-2 | β=-1.5 | δ=0.1 | δ=0.25 | δ=0.5 |
|  | 0.83 | 0.23 | 0.16 | 0.71 | 0.59 | 0.39 | 0.17 | 0.06 |
|  | **Leader-Centrality Emergence** | | | | | | | |
|  | **No PP** | **PL-decay** | | **Exp-decay** | | **Social** | | |
| **Centrality** |  | α=-2 | α=-1.5 | β=-2 | β=-1.5 | δ=0.1 | δ=0.25 | δ=0.5 |
| BC | 0.81 | 0.21 | 0.14 | 0.73 | 0.59 | 0.37 | 0.16 | 0.05 |
| CC | 0.98 | 0.24 | 0.16 | 0.87 | 0.66 | 0.40 | 0.16 | 0.05 |
| DC | 0.98 | 0.23 | 0.16 | 0.84 | 0.64 | 0.41 | 0.17 | 0.05 |
| EC | 1.00 | 0.23 | 0.17 | 0.86 | 0.65 | 0.41 | 0.17 | 0.06 |
| SC | 1.00 | 0.24 | 0.17 | 0.87 | 0.68 | 0.41 | 0.17 | 0.06 |

Supplementary Table S21

Normalized consensus times for the HS network with divergence of 0.1.

|  | **Random Emergence** | | | | | | | |
| --- | --- | --- | --- | --- | --- | --- | --- | --- |
|  | **No PP** | **PL-decay** | | **Exp-decay** | | **Social** | | |
|  |  | α=-2 | α=-1.5 | β=-2 | β=-1.5 | δ=0.1 | δ=0.25 | δ=0.5 |
|  | 0.75 | 0.20 | 0.16 | 0.69 | 0.51 | 0.35 | 0.18 | 0.07 |
|  | **Leader-Centrality Emergence** | | | | | | | |
|  | **No PP** | **PL-decay** | | **Exp-decay** | | **Social** | | |
| **Centrality** |  | α=-2 | α=-1.5 | β=-2 | β=-1.5 | δ=0.1 | δ=0.25 | δ=0.5 |
| BC | 0.65 | 0.18 | 0.14 | 0.56 | 0.43 | 0.28 | 0.15 | 0.06 |
| CC | 0.99 | 0.20 | 0.13 | 0.81 | 0.60 | 0.35 | 0.14 | 0.06 |
| DC | 0.69 | 0.17 | 0.11 | 0.59 | 0.46 | 0.27 | 0.13 | 0.07 |
| EC | 0.99 | 0.21 | 0.15 | 0.81 | 0.57 | 0.34 | 0.14 | 0.04 |
| SC | 1.00 | 0.21 | 0.15 | 0.78 | 0.59 | 0.34 | 0.14 | 0.04 |

Supplementary Table S22

Normalized consensus times for the High Tech network with divergence of 0.1.

|  | **Random Emergence** | | | | | | | |
| --- | --- | --- | --- | --- | --- | --- | --- | --- |
|  | **No PP** | **PL-decay** | | **Exp-decay** | | **Social** | | |
|  |  | α=-2 | α=-1.5 | β=-2 | β=-1.5 | δ=0.1 | δ=0.25 | δ=0.5 |
|  | 0.93 | 0.25 | 0.19 | 0.83 | 0.67 | 0.41 | 0.18 | 0.07 |
|  | **Leader-Centrality Emergence** | | | | | | | |
|  | **No PP** | **PL-decay** | | **Exp-decay** | | **Social** | | |
| **Centrality** |  | α=-2 | α=-1.5 | β=-2 | β=-1.5 | δ=0.1 | δ=0.25 | δ=0.5 |
| BC | 0.78 | 0.22 | 0.16 | 0.70 | 0.56 | 0.37 | 0.16 | 0.06 |
| CC | 0.97 | 0.24 | 0.17 | 0.84 | 0.66 | 0.41 | 0.18 | 0.06 |
| DC | 0.93 | 0.24 | 0.18 | 0.83 | 0.67 | 0.40 | 0.18 | 0.06 |
| EC | 0.98 | 0.25 | 0.19 | 0.88 | 0.68 | 0.42 | 0.19 | 0.07 |
| SC | 1.00 | 0.27 | 0.19 | 0.88 | 0.68 | 0.44 | 0.19 | 0.06 |

Supplementary Table S23

Normalized consensus times for the Math Method network with divergence of 0.1.

|  | **Random Emergence** | | | | | | | |
| --- | --- | --- | --- | --- | --- | --- | --- | --- |
|  | **No PP** | **PL-decay** | | **Exp-decay** | | **Social** | | |
|  |  | α=-2 | α=-1.5 | β=-2 | β=-1.5 | δ=0.1 | δ=0.25 | δ=0.5 |
|  | 0.87 | 0.26 | 0.19 | 0.77 | 0.61 | 0.41 | 0.19 | 0.07 |
|  | **Leader-Centrality Emergence** | | | | | | | |
|  | **No PP** | **PL-decay** | | **Exp-decay** | | **Social** | | |
| **Centrality** |  | α=-2 | α=-1.5 | β=-2 | β=-1.5 | δ=0.1 | δ=0.25 | δ=0.5 |
| BC | 0.63 | 0.22 | 0.16 | 0.59 | 0.50 | 0.35 | 0.17 | 0.06 |
| CC | 1.00 | 0.26 | 0.19 | 0.87 | 0.71 | 0.47 | 0.19 | 0.07 |
| DC | 0.98 | 0.26 | 0.18 | 0.86 | 0.70 | 0.46 | 0.19 | 0.07 |
| EC | 0.96 | 0.27 | 0.18 | 0.90 | 0.74 | 0.45 | 0.20 | 0.07 |
| SC | 1.00 | 0.26 | 0.19 | 0.86 | 0.71 | 0.44 | 0.20 | 0.07 |

Supplementary Table S24

Normalized consensus times for the Sawmill network with divergence of 0.1.

|  | **Random Emergence** | | | | | | | |
| --- | --- | --- | --- | --- | --- | --- | --- | --- |
|  | **No PP** | **PL-decay** | | **Exp-decay** | | **Social** | | |
|  |  | α=-2 | α=-1.5 | β=-2 | β=-1.5 | δ=0.1 | δ=0.25 | δ=0.5 |
|  | 1.00 | 0.24 | 0.17 | 0.88 | 0.68 | 0.45 | 0.20 | 0.07 |
|  | **Leader-Centrality Emergence** | | | | | | | |
|  | **No PP** | **PL-decay** | | **Exp-decay** | | **Social** | | |
| **Centrality** |  | α=-2 | α=-1.5 | β=-2 | β=-1.5 | δ=0.1 | δ=0.25 | δ=0.5 |
| BC | 0.72 | 0.19 | 0.13 | 0.65 | 0.54 | 0.38 | 0.17 | 0.05 |
| CC | 0.75 | 0.19 | 0.12 | 0.70 | 0.56 | 0.40 | 0.17 | 0.05 |
| DC | 0.74 | 0.19 | 0.12 | 0.71 | 0.59 | 0.40 | 0.17 | 0.05 |
| EC | 0.99 | 0.22 | 0.15 | 0.92 | 0.68 | 0.45 | 0.18 | 0.05 |
| SC | 0.82 | 0.20 | 0.13 | 0.76 | 0.62 | 0.41 | 0.18 | 0.05 |

Supplementary Table S25

Normalized consensus times for the Social3 network with divergence of 0.1.

|  | **Random Emergence** | | | | | | | |
| --- | --- | --- | --- | --- | --- | --- | --- | --- |
|  | **No PP** | **PL-decay** | | **Exp-decay** | | **Social** | | |
|  |  | α=-2 | α=-1.5 | β=-2 | β=-1.5 | δ=0.1 | δ=0.25 | δ=0.5 |
|  | 0.92 | 0.33 | 0.24 | 0.81 | 0.67 | 0.46 | 0.24 | 0.11 |
|  | **Leader-Centrality Emergence** | | | | | | | |
|  | **No PP** | **PL-decay** | | **Exp-decay** | | **Social** | | |
| **Centrality** |  | α=-2 | α=-1.5 | β=-2 | β=-1.5 | δ=0.1 | δ=0.25 | δ=0.5 |
| BC | 0.92 | 0.31 | 0.24 | 0.79 | 0.68 | 0.42 | 0.21 | 0.07 |
| CC | 0.98 | 0.32 | 0.23 | 0.84 | 0.70 | 0.45 | 0.22 | 0.08 |
| DC | 1.00 | 0.33 | 0.25 | 0.87 | 0.70 | 0.45 | 0.23 | 0.08 |
| EC | 0.96 | 0.32 | 0.24 | 0.88 | 0.74 | 0.46 | 0.23 | 0.08 |
| SC | 0.92 | 0.32 | 0.25 | 0.86 | 0.69 | 0.46 | 0.22 | 0.09 |

Supplementary Table S26

Normalized consensus times for the BA random graph with divergence of 0.2.

|  | **Random Emergence** | | | | | | | |
| --- | --- | --- | --- | --- | --- | --- | --- | --- |
|  | **No PP** | **PL-decay** | | **Exp-decay** | | **Social** | | |
|  |  | α=-2 | α=-1.5 | β=-2 | β=-1.5 | δ=0.1 | δ=0.25 | δ=0.5 |
|  | 1.00 | 0.42 | 0.29 | 0.94 | 0.85 | 0.79 | 0.33 | 0.11 |
|  | **Leader-Centrality Emergence** | | | | | | | |
|  | **No PP** | **PL-decay** | | **Exp-decay** | | **Social** | | |
| **Centrality** |  | α=-2 | α=-1.5 | β=-2 | β=-1.5 | δ=0.1 | δ=0.25 | δ=0.5 |
| BC | 0.69 | 0.28 | 0.22 | 0.60 | 0.51 | 0.34 | 0.18 | 0.08 |
| CC | 0.74 | 0.29 | 0.22 | 0.64 | 0.54 | 0.35 | 0.18 | 0.08 |
| DC | 0.70 | 0.28 | 0.22 | 0.60 | 0.50 | 0.33 | 0.19 | 0.08 |
| EC | 0.72 | 0.28 | 0.23 | 0.63 | 0.53 | 0.34 | 0.18 | 0.07 |
| SC | 0.71 | 0.28 | 0.22 | 0.62 | 0.53 | 0.34 | 0.18 | 0.08 |

Supplementary Table S27

Normalized consensus times for the ER random graph with divergence of 0.2.

|  | **Random Emergence** | | | | | | | |
| --- | --- | --- | --- | --- | --- | --- | --- | --- |
|  | **No PP** | **PL-decay** | | **Exp-decay** | | **Social** | | |
|  |  | α=-2 | α=-1.5 | β=-2 | β=-1.5 | δ=0.1 | δ=0.25 | δ=0.5 |
|  | 1.00 | 0.27 | 0.18 | 0.88 | 0.70 | 0.45 | 0.20 | 0.07 |
|  | **Leader-Centrality Emergence** | | | | | | | |
|  | **No PP** | **PL-decay** | | **Exp-decay** | | **Social** | | |
| **Centrality** |  | α=-2 | α=-1.5 | β=-2 | β=-1.5 | δ=0.1 | δ=0.25 | δ=0.5 |
| BC | 0.67 | 0.21 | 0.16 | 0.57 | 0.46 | 0.33 | 0.18 | 0.07 |
| CC | 0.78 | 0.22 | 0.14 | 0.67 | 0.54 | 0.32 | 0.14 | 0.03 |
| DC | 0.70 | 0.22 | 0.18 | 0.58 | 0.45 | 0.36 | 0.20 | 0.08 |
| EC | 0.74 | 0.20 | 0.14 | 0.63 | 0.50 | 0.32 | 0.15 | 0.05 |
| SC | 0.75 | 0.23 | 0.17 | 0.64 | 0.51 | 0.36 | 0.18 | 0.07 |

Supplementary Table S28

Normalized consensus times for the Corporate network with divergence of 0.2.

|  | **Random Emergence** | | | | | | | |
| --- | --- | --- | --- | --- | --- | --- | --- | --- |
|  | **No PP** | **PL-decay** | | **Exp-decay** | | **Social** | | |
|  |  | α=-2 | α=-1.5 | β=-2 | β=-1.5 | δ=0.1 | δ=0.25 | δ=0.5 |
|  | 1.00 | 0.19 | 0.11 | 0.78 | 0.64 | 0.45 | 0.17 | 0.04 |
|  | **Leader-Centrality Emergence** | | | | | | | |
|  | **No PP** | **PL-decay** | | **Exp-decay** | | **Social** | | |
| **Centrality** |  | α=-2 | α=-1.5 | β=-2 | β=-1.5 | δ=0.1 | δ=0.25 | δ=0.5 |
| BC | 0.22 | 0.01 | 0.00 | 0.17 | 0.11 | 0.09 | 0.03 | 0.002 |
| CC | 0.28 | 0.02 | 0.02 | 0.23 | 0.15 | 0.11 | 0.03 | 0.002 |
| DC | 0.24 | 0.04 | 0.03 | 0.20 | 0.13 | 0.12 | 0.04 | 0.007 |
| EC | 0.22 | 0.01 | 0.00 | 0.18 | 0.11 | 0.09 | 0.03 | 0.002 |
| SC | 0.23 | 0.01 | 0.00 | 0.18 | 0.11 | 0.09 | 0.03 | 0.002 |

Supplementary Table S29

Normalized consensus times for the Drugs network with divergence of 0.2.

|  | **Random Emergence** | | | | | | | |
| --- | --- | --- | --- | --- | --- | --- | --- | --- |
|  | **No PP** | **PL-decay** | | **Exp-decay** | | **Social** | | |
|  |  | α=-2 | α=-1.5 | β=-2 | β=-1.5 | δ=0.1 | δ=0.25 | δ=0.5 |
|  | 1.00 | 0.15 | 0.09 | 0.76 | 0.45 | 0.44 | 0.19 | 0.05 |
|  | **Leader-Centrality Emergence** | | | | | | | |
|  | **No PP** | **PL-decay** | | **Exp-decay** | | **Social** | | |
| **Centrality** |  | α=-2 | α=-1.5 | β=-2 | β=-1.5 | δ=0.1 | δ=0.25 | δ=0.5 |
| BC | 0.69 | 0.06 | 0.04 | 0.52 | 0.34 | 0.25 | 0.06 | 0.02 |
| CC | 0.47 | 0.07 | 0.05 | 0.44 | 0.30 | 0.23 | 0.07 | 0.01 |
| DC | 0.46 | 0.04 | 0.05 | 0.37 | 0.24 | 0.20 | 0.08 | 0.04 |
| EC | 0.51 | 0.08 | 0.06 | 0.42 | 0.31 | 0.21 | 0.08 | 0.03 |
| SC | 0.51 | 0.08 | 0.06 | 0.40 | 0.32 | 0.21 | 0.08 | 0.03 |

Supplementary Table S30

Normalized consensus times for the Prison network with divergence of 0.2.

|  | **Random Emergence** | | | | | | | |
| --- | --- | --- | --- | --- | --- | --- | --- | --- |
|  | **No PP** | **PL-decay** | | **Exp-decay** | | **Social** | | |
|  |  | α=-2 | α=-1.5 | β=-2 | β=-1.5 | δ=0.1 | δ=0.25 | δ=0.5 |
|  | 1.00 | 0.29 | 0.20 | 0.91 | 0.73 | 0.50 | 0.24 | 0.08 |
|  | **Leader-Centrality Emergence** | | | | | | | |
|  | **No PP** | **PL-decay** | | **Exp-decay** | | **Social** | | |
| **Centrality** |  | α=-2 | α=-1.5 | β=-2 | β=-1.5 | δ=0.1 | δ=0.25 | δ=0.5 |
| BC | 0.81 | 0.15 | 0.10 | 0.74 | 0.56 | 0.38 | 0.15 | 0.03 |
| CC | 0.83 | 0.15 | 0.09 | 0.73 | 0.57 | 0.39 | 0.15 | 0.04 |
| DC | 0.82 | 0.15 | 0.09 | 0.83 | 0.55 | 0.39 | 0.14 | 0.05 |
| EC | 0.90 | 0.15 | 0.10 | 0.79 | 0.58 | 0.39 | 0.15 | 0.03 |
| SC | 0.85 | 0.15 | 0.09 | 0.74 | 0.56 | 0.39 | 0.14 | 0.05 |

Supplementary Table S31

Normalized consensus times for the Zachary network with divergence of 0.2.

|  | **Random Emergence** | | | | | | | |
| --- | --- | --- | --- | --- | --- | --- | --- | --- |
|  | **No PP** | **PL-decay** | | **Exp-decay** | | **Social** | | |
|  |  | α=-2 | α=-1.5 | β=-2 | β=-1.5 | δ=0.1 | δ=0.25 | δ=0.5 |
|  | 1.00 | 0.27 | 0.21 | 0.83 | 0.64 | 0.40 | 0.19 | 0.08 |
|  | **Leader-Centrality Emergence** | | | | | | | |
|  | **No PP** | **PL-decay** | | **Exp-decay** | | **Social** | | |
| **Centrality** |  | α=-2 | α=-1.5 | β=-2 | β=-1.5 | δ=0.1 | δ=0.25 | δ=0.5 |
| BC | 0.72 | 0.23 | 0.18 | 0.62 | 0.49 | 0.30 | 0.14 | 0.07 |
| CC | 0.72 | 0.23 | 0.18 | 0.61 | 0.48 | 0.30 | 0.14 | 0.06 |
| DC | 0.72 | 0.22 | 0.17 | 0.60 | 0.47 | 0.29 | 0.16 | 0.06 |
| EC | 0.70 | 0.22 | 0.17 | 0.59 | 0.48 | 0.31 | 0.15 | 0.05 |
| SC | 0.73 | 0.22 | 0.18 | 0.59 | 0.48 | 0.29 | 0.15 | 0.06 |

Supplementary Table S32

Normalized consensus times for the Colorado Springs network with divergence of 0.2.

|  | **Random Emergence** | | | | | | | |
| --- | --- | --- | --- | --- | --- | --- | --- | --- |
|  | **No PP** | **PL-decay** | | **Exp-decay** | | **Social** | | |
|  |  | α=-2 | α=-1.5 | β=-2 | β=-1.5 | δ=0.1 | δ=0.25 | δ=0.5 |
|  | 0.70 | 0.27 | 0.16 | 0.63 | 0.57 | 0.64 | 0.40 | 0.11 |
|  | **Leader-Centrality Emergence** | | | | | | | |
|  | **No PP** | **PL-decay** | | **Exp-decay** | | **Social** | | |
| **Centrality** |  | α=-2 | α=-1.5 | β=-2 | β=-1.5 | δ=0.1 | δ=0.25 | δ=0.5 |
| BC | 1.00 | 0.11 | 0.07 | 0.86 | 0.63 | 0.50 | 0.20 | 0.04 |
| CC | 0.84 | 0.07 | 0.04 | 0.74 | 0.58 | 0.49 | 0.21 | 0.03 |
| DC | 0.81 | 0.13 | 0.09 | 0.73 | 0.56 | 0.43 | 0.20 | 0.05 |
| EC | 0.71 | 0.17 | 0.09 | 0.62 | 0.46 | 0.34 | 0.16 | 0.03 |
| SC | 0.91 | 0.17 | 0.11 | 0.85 | 0.64 | 0.49 | 0.24 | 0.06 |

Supplementary Table S33

Normalized consensus times for the Dolphins network with divergence of 0.2.

|  | **Random Emergence** | | | | | | | |
| --- | --- | --- | --- | --- | --- | --- | --- | --- |
|  | **No PP** | **PL-decay** | | **Exp-decay** | | **Social** | | |
|  |  | α=-2 | α=-1.5 | β=-2 | β=-1.5 | δ=0.1 | δ=0.25 | δ=0.5 |
|  | 0.84 | 0.24 | 0.18 | 0.76 | 0.59 | 0.41 | 0.19 | 0.08 |
|  | **Leader-Centrality Emergence** | | | | | | | |
|  | **No PP** | **PL-decay** | | **Exp-decay** | | **Social** | | |
| **Centrality** |  | α=-2 | α=-1.5 | β=-2 | β=-1.5 | δ=0.1 | δ=0.25 | δ=0.5 |
| BC | 0.72 | 0.16 | 0.11 | 0.65 | 0.52 | 0.32 | 0.14 | 0.06 |
| CC | 0.92 | 0.16 | 0.10 | 0.82 | 0.60 | 0.40 | 0.14 | 0.05 |
| DC | 0.67 | 0.15 | 0.10 | 0.63 | 0.50 | 0.34 | 0.14 | 0.06 |
| EC | 0.94 | 0.19 | 0.12 | 0.84 | 0.63 | 0.41 | 0.17 | 0.05 |
| SC | 1.00 | 0.18 | 0.12 | 0.84 | 0.65 | 0.45 | 0.17 | 0.06 |

Supplementary Table S34

Normalized consensus times for the Galesburg network with divergence of 0.2.

|  | **Random Emergence** | | | | | | | |
| --- | --- | --- | --- | --- | --- | --- | --- | --- |
|  | **No PP** | **PL-decay** | | **Exp-decay** | | **Social** | | |
|  |  | α=-2 | α=-1.5 | β=-2 | β=-1.5 | δ=0.1 | δ=0.25 | δ=0.5 |
|  | 0.81 | 0.24 | 0.18 | 0.71 | 0.56 | 0.38 | 0.18 | 0.07 |
|  | **Leader-Centrality Emergence** | | | | | | | |
|  | **No PP** | **PL-decay** | | **Exp-decay** | | **Social** | | |
| **Centrality** |  | α=-2 | α=-1.5 | β=-2 | β=-1.5 | δ=0.1 | δ=0.25 | δ=0.5 |
| BC | 0.80 | 0.20 | 0.15 | 0.70 | 0.56 | 0.36 | 0.15 | 0.06 |
| CC | 0.93 | 0.22 | 0.16 | 0.83 | 0.62 | 0.39 | 0.16 | 0.05 |
| DC | 0.94 | 0.22 | 0.16 | 0.83 | 0.64 | 0.39 | 0.16 | 0.06 |
| EC | 0.97 | 0.23 | 0.18 | 0.85 | 0.65 | 0.41 | 0.16 | 0.06 |
| SC | 1.00 | 0.24 | 0.17 | 0.84 | 0.64 | 0.40 | 0.17 | 0.06 |

Supplementary Table S35

Normalized consensus times for the HS network with divergence of 0.2

|  | **Random Emergence** | | | | | | | |
| --- | --- | --- | --- | --- | --- | --- | --- | --- |
|  | **No PP** | **PL-decay** | | **Exp-decay** | | **Social** | | |
|  |  | α=-2 | α=-1.5 | β=-2 | β=-1.5 | δ=0.1 | δ=0.25 | δ=0.5 |
|  | 0.76 | 0.23 | 0.17 | 0.65 | 0.51 | 0.37 | 0.20 | 0.08 |
|  | **Leader-Centrality Emergence** | | | | | | | |
|  | **No PP** | **PL-decay** | | **Exp-decay** | | **Social** | | |
| **Centrality** |  | α=-2 | α=-1.5 | β=-2 | β=-1.5 | δ=0.1 | δ=0.25 | δ=0.5 |
| BC | 0.60 | 0.20 | 0.16 | 0.51 | 0.39 | 0.27 | 0.16 | 0.07 |
| CC | 0.99 | 0.21 | 0.16 | 0.83 | 0.60 | 0.34 | 0.15 | 0.06 |
| DC | 0.66 | 0.14 | 0.11 | 0.53 | 0.43 | 0.25 | 0.13 | 0.07 |
| EC | 0.99 | 0.23 | 0.17 | 0.82 | 0.62 | 0.35 | 0.15 | 0.05 |
| SC | 1.00 | 0.24 | 0.17 | 0.81 | 0.60 | 0.34 | 0.15 | 0.05 |

Supplementary Table S36

Normalized consensus times for the High Tech network with divergence of 0.2.

|  | **Random Emergence** | | | | | | | |
| --- | --- | --- | --- | --- | --- | --- | --- | --- |
|  | **No PP** | **PL-decay** | | **Exp-decay** | | **Social** | | |
|  |  | α=-2 | α=-1.5 | β=-2 | β=-1.5 | δ=0.1 | δ=0.25 | δ=0.5 |
|  | 0.93 | 0.28 | 0.22 | 0.81 | 0.65 | 0.42 | 0.20 | 0.08 |
|  | **Leader-Centrality Emergence** | | | | | | | |
|  | **No PP** | **PL-decay** | | **Exp-decay** | | **Social** | | |
| **Centrality** |  | α=-2 | α=-1.5 | β=-2 | β=-1.5 | δ=0.1 | δ=0.25 | δ=0.5 |
| BC | 0.76 | 0.22 | 0.17 | 0.69 | 0.56 | 0.35 | 0.16 | 0.06 |
| CC | 0.94 | 0.25 | 0.18 | 0.81 | 0.64 | 0.40 | 0.17 | 0.05 |
| DC | 0.95 | 0.24 | 0.19 | 0.82 | 0.66 | 0.40 | 0.17 | 0.07 |
| EC | 0.97 | 0.27 | 0.20 | 0.84 | 0.67 | 0.41 | 0.18 | 0.07 |
| SC | 1.00 | 0.27 | 0.20 | 0.86 | 0.69 | 0.42 | 0.18 | 0.06 |

Supplementary Table S37

Normalized consensus times for the Math Method network with divergence of 0.2.

|  | **Random Emergence** | | | | | | | |
| --- | --- | --- | --- | --- | --- | --- | --- | --- |
|  | **No PP** | **PL-decay** | | **Exp-decay** | | **Social** | | |
|  |  | α=-2 | α=-1.5 | β=-2 | β=-1.5 | δ=0.1 | δ=0.25 | δ=0.5 |
|  | 0.79 | 0.25 | 0.19 | 0.76 | 0.62 | 0.43 | 0.20 | 0.08 |
|  | **Leader-Centrality Emergence** | | | | | | | |
|  | **No PP** | **PL-decay** | | **Exp-decay** | | **Social** | | |
| **Centrality** |  | α=-2 | α=-1.5 | β=-2 | β=-1.5 | δ=0.1 | δ=0.25 | δ=0.5 |
| BC | 0.60 | 0.23 | 0.18 | 0.56 | 0.49 | 0.32 | 0.16 | 0.06 |
| CC | 1.00 | 0.26 | 0.18 | 0.90 | 0.68 | 0.43 | 0.19 | 0.07 |
| DC | 0.98 | 0.25 | 0.18 | 0.87 | 0.69 | 0.42 | 0.19 | 0.07 |
| EC | 0.96 | 0.25 | 0.18 | 0.85 | 0.71 | 0.42 | 0.20 | 0.06 |
| SC | 0.93 | 0.25 | 0.18 | 0.85 | 0.70 | 0.43 | 0.19 | 0.06 |

Supplementary Table S38

Normalized consensus times for the Sawmill network with divergence of 0.2.

|  | **Random Emergence** | | | | | | | |
| --- | --- | --- | --- | --- | --- | --- | --- | --- |
|  | **No PP** | **PL-decay** | | **Exp-decay** | | **Social** | | |
|  |  | α=-2 | α=-1.5 | β=-2 | β=-1.5 | δ=0.1 | δ=0.25 | δ=0.5 |
|  | 0.96 | 0.23 | 0.16 | 0.87 | 0.66 | 0.43 | 0.19 | 0.06 |
|  | **Leader-Centrality Emergence** | | | | | | | |
|  | **No PP** | **PL-decay** | | **Exp-decay** | | **Social** | | |
| **Centrality** |  | α=-2 | α=-1.5 | β=-2 | β=-1.5 | δ=0.1 | δ=0.25 | δ=0.5 |
| BC | 0.63 | 0.17 | 0.12 | 0.59 | 0.46 | 0.33 | 0.14 | 0.05 |
| CC | 0.66 | 0.16 | 0.12 | 0.58 | 0.49 | 0.33 | 0.14 | 0.04 |
| DC | 0.64 | 0.15 | 0.10 | 0.60 | 0.48 | 0.33 | 0.14 | 0.04 |
| EC | 1.00 | 0.19 | 0.13 | 0.85 | 0.63 | 0.37 | 0.15 | 0.05 |
| SC | 0.75 | 0.17 | 0.12 | 0.65 | 0.51 | 0.35 | 0.14 | 0.04 |

Supplementary Table S39

Normalized consensus times for the Social3 network with divergence of 0.2.

|  | **Random Emergence** | | | | | | | |
| --- | --- | --- | --- | --- | --- | --- | --- | --- |
|  | **No PP** | **PL-decay** | | **Exp-decay** | | **Social** | | |
|  |  | α=-2 | α=-1.5 | β=-2 | β=-1.5 | δ=0.1 | δ=0.25 | δ=0.5 |
|  | 0.94 | 0.37 | 0.29 | 0.83 | 0.71 | 0.50 | 0.28 | 0.13 |
|  | **Leader-Centrality Emergence** | | | | | | | |
|  | **No PP** | **PL-decay** | | **Exp-decay** | | **Social** | | |
| **Centrality** |  | α=-2 | α=-1.5 | β=-2 | β=-1.5 | δ=0.1 | δ=0.25 | δ=0.5 |
| BC | 0.90 | 0.35 | 0.27 | 0.83 | 0.70 | 0.43 | 0.23 | 0.08 |
| CC | 0.97 | 0.34 | 0.27 | 0.87 | 0.73 | 0.45 | 0.22 | 0.09 |
| DC | 0.90 | 0.36 | 0.27 | 0.87 | 0.72 | 0.45 | 0.22 | 0.08 |
| EC | 1.00 | 0.31 | 0.25 | 0.87 | 0.73 | 0.44 | 0.23 | 0.09 |
| SC | 0.94 | 0.33 | 0.24 | 0.83 | 0.70 | 0.44 | 0.21 | 0.11 |

Supplementary References

Lancichinetti, A., Fortunato, S. & Radicchi, F. Benchmark graphs for testing community detection algorithms. *Phys. Rev. E*, **78**, 046110 (2008).

De Nooy, W., Mrvar, A. & Batagelj, V. *Exploratory Social Network Analysis with Pajek* (Cambridge University Press, Cambridge, 2004).

Herzog, W. A., Stanfield, J. D., Whiting, G. C. & Svenning, L. *Patterns of Diffusion in Rural Brazil* (Research Report of Phase II, Michigan State University, 1968).

Valente, T. W. *Empirical Networks Project* (December 2012). <http://www-hsc.usc.edu/~tvalente/enp/enp.doc>.
